# Targeting Oxidative Stress for Treatment of Glaucoma and Optic Neuritis

**DOI:** 10.1155/2017/2817252

**Published:** 2017-02-08

**Authors:** Atsuko Kimura, Kazuhiko Namekata, Xiaoli Guo, Takahiko Noro, Chikako Harada, Takayuki Harada

**Affiliations:** Visual Research Project, Tokyo Metropolitan Institute of Medical Science, Tokyo, Japan

## Abstract

Glaucoma is a neurodegenerative disease of the eye and it is one of the leading causes of blindness. Glaucoma is characterized by progressive degeneration of retinal ganglion cells (RGCs) and their axons, namely, the optic nerve, usually associated with elevated intraocular pressure (IOP). Current glaucoma therapies target reduction of IOP, but since RGC death is the cause of irreversible vision loss, neuroprotection may be an effective strategy for glaucoma treatment. One of the risk factors for glaucoma is increased oxidative stress, and drugs with antioxidative properties including valproic acid and spermidine, as well as inhibition of apoptosis signal-regulating kinase 1, an enzyme that is involved in oxidative stress, have been reported to prevent glaucomatous retinal degeneration in mouse models of glaucoma. Optic neuritis is a demyelinating inflammation of the optic nerve that presents with visual impairment and it is commonly associated with multiple sclerosis, a chronic demyelinating disease of the central nervous system. Although steroids are commonly used for treatment of optic neuritis, reduction of oxidative stress by approaches such as gene therapy is effective in ameliorating optic nerve demyelination in preclinical studies. In this review, we discuss oxidative stress as a therapeutic target for glaucoma and optic neuritis.

## 1. Introduction

Glaucoma is a neurodegenerative disease of the eye and it is one of the major causes of irreversible blindness. It is estimated that, by 2020, more than 80 million people will be affected worldwide, with at least 6 to 8 million of them becoming bilaterally blind [1]. Glaucoma is characterized by damage to the optic nerve and progressive degeneration of retinal ganglion cells (RGCs), which are critical elements for vision loss. The factors associated with pathogenesis of glaucoma include high intraocular pressure (IOP), increased oxidative stress, aging, glutamate neurotoxicity, and susceptibility genes such as optineurin and myocilin [2–4].

Optic neuritis is a demyelinating inflammation of the optic nerve and it typically affects young adults ranging from 18 to 45 years of age. Patients usually present with an acute reduction of visual acuity, orbital pain exacerbated by eye movements, dyschromatopsia, and an afferent papillary defect, with or without swelling of the optic nerve head. There is a strong association between optic neuritis and multiple sclerosis (MS), an acute inflammatory demyelinating disease of the central nervous system (CNS), in which optic neuritis is the initial presentation of MS for approximately 20% of MS patients and a risk of developing MS by 15 years after the onset of optic neuritis is 50% [5]. Research into optic neuritis is somewhat limited compared with MS research, but it is an important area of research that is continuously making progress.

In this review, we discuss the role of oxidative stress in the pathogenesis of glaucoma and optic neuritis and how we can target oxidative stress for treatment of these two disease conditions.

## 2. Oxidative Stress and Glaucoma

Oxidative stress reflects an imbalance between the production of reactive oxygen species (free radicals) and antioxidant defenses, in which oxidative processes exceed antioxidant systems. Oxidative stress is an important risk factor in human glaucoma [6] and consistently, the plasma level of glutathione (GSH), an important antioxidant, is decreased in glaucoma patients [7, 8]. Normal tension glaucoma (NTG) is a subtype of glaucoma that does not present with high IOP and there is an unexpectedly high prevalence of NTG in Japan and other Asian countries [9, 10]. Previously, we reported spontaneous mouse models of NTG; these mice lacked the glutamate transporter genes* EAAC1* and* GLAST*, in which EAAC1 is expressed in neurons and GLAST is expressed in Müller glia in the retina [11]. These mice exhibit spontaneous RGC death and optic nerve degeneration without an increase in IOP, a pathology that is similar to NTG. Glutamate transporters clear excess glutamate from the synapse, thus preventing excitotoxic damage on surrounding retinal neurons [12]. In addition, glutamate that is transported into cells by the glutamate uptake process, together with cysteine and glycine, is converted to GSH, a major antioxidant in the retina [13]. Therefore, glutamate transporters play important roles in reducing excitotoxic and oxidative stress damage to cells. To this end, GLAST KO mice and EAAC1 KO mice exhibit the key pathological features of NTG as a result of increased glutamate neurotoxicity and oxidative stress. These mice have been useful in providing important information on therapeutic targets for NTG [14–23].

Although glaucoma therapy that is currently available focuses on reduction of IOP, some patients do not respond to this type of treatment and research into neuroprotection of RGCs as a novel therapeutic strategy is advancing. One of such strategies is reduction of oxidative stress [4]. For example, an antioxidant *α*-lipoic acid protects RGCs in the glaucomatous retina in DBA/2J mice, an animal model that recapitulates the slow and progressive nature of human glaucoma [24, 25], and administration of another antioxidant, tempol, reduces RGC death in an experimental glaucoma model [26]. Furthermore, geranylgeranylacetone (GGA), which is used for treatment of gastric ulcers, can act as an antioxidant by directly inducing the cytoprotective heat shock protein 70 (Hsp70) expression and inhibits cell apoptosis caused by H_2_O_2_ in cultured hepatocytes [27]. It also reduces oxidative stress levels following light-induced retinal damage [28] and increases survival of retinal neurons in an ischemic retinal injury model [29]. Oral administration of GGA induces Hsp70 expression in the retina and suppresses RGC death in GLAST KO mice, a mouse model of NTG [22]. Therefore, targeting to reduce oxidative stress in the retina may be a novel therapeutic strategy for glaucoma.

## 3. Inhibition of Oxidative Stress for Treatment of Glaucoma

The summary of this section is shown in Table 1.

### 3.1. Apoptosis Signal-Regulating Kinase 1 (ASK1)

Apoptosis signal-regulating kinase 1 (ASK1) is a member of mitogen-activated protein kinase kinase kinase (MAP3K) that plays key roles in cellular responses to oxidative stress and endoplasmic reticulum stress [30, 31]. ASK1 acts downstream of tumor necrosis factor alpha (TNF-*α*) signalling and is a key regulator of stress- and cytokine-induced apoptosis [32]. It has been reported that stress such as serum withdrawal or TNF-*α* generates ROS that activates ASK1 by removing a physiological inhibitor of ASK1, thioredoxin, and initiates the ASK1-mediated apoptotic pathway [33]. ASK1 is strongly activated in response to various oxidants such as H_2_O_2_ and the activation of the ASK1-JNK/p38 pathway plays an essential part in oxidative stress-induced apoptosis [34]. We have previously reported that deletion of the* ASK1* gene prevents RGC death in various mouse models of glaucoma, including retinal ischemia, optic nerve injury (ONI), and GLAST KO mice (GLAST/ASK1 double KO mice) [15, 35, 36]. In all the models we have used, ASK1 deficiency reduced oxidative stress levels that led to increased RGC survival, indicating that targeting oxidative stress is an effective approach for treatment of glaucoma. It is important to note that the therapeutic effect of ASK1 deletion may also involve reduction of factors that cause oxidative stress, such as TNF-*α* [37, 38], which mediates neurodegeneration in glaucoma [39]. Currently, we are examining if a therapeutic effect is achieved by oral administration of an ASK1 inhibitor in EAAC1 KO mice, a spontaneous mouse model of NTG [11], to further confirm that ASK1 inhibition is a promising target for treatment of glaucoma.

### 3.2. Dedicator of Cytokinesis 3 (Dock3)

Dedicator of cytokinesis 3 (Dock3) belongs to a family of atypical guanine exchange factors (GEFs). It is specifically expressed in the CNS and regulates actin cytoskeleton dynamics causing cellular morphological changes by activating the small GTPase Rac1 [40, 41]. Recent studies have indicated that Dock3 acts downstream of the brain-derived neurotrophic factor- (BDNF-) TrkB pathway [42] and possesses functions that are independent of its GEF activity: for example, it directly binds to GSK-3*β* and stimulates microtubule dynamics to promote optic nerve regeneration [43, 44]. Interestingly, Dock3 also binds to GluN2B, one of the subunits for* N*-methyl-D-aspartate (NMDA) receptors, and reduces the NMDA receptor expression leading to RGC protection from NMDA-induced cell death and in GLAST KO mice [16]. Stimulation of NMDA receptors leads to superoxide production and neurotoxicity in neurons [45, 46]. Therefore, it is possible that Dock3 reduces oxidative stress indirectly by attenuating NMDA receptor activation. In addition, overexpression of Dock3 in cultured RGCs increases cell survival following H_2_O_2_ stimulation and in vivo, the activation of the ASK1-p38 pathway is decreased in mice with Dock3 overexpression following ONI [16, 47]. These results suggest the possibility that Dock3 prevents oxidative stress-induced RGC death by suppression of the ASK1 pathway. Further studies are required to confirm this.

### 3.3. Valproic Acid (VPA)

Valproic acid (VPA) is a short chain fatty acid that has been used clinically worldwide for treatment of epilepsy since the 1970s. It exerts multiple pharmacological actions and one of the recently identified effects is inhibition of histone deacetylases, which is distinct from its therapeutic antiepileptic activity [48, 49]. Recently, we reported that VPA prevents glaucoma-like retinal degeneration in mouse models of glaucoma, by inhibition of the oxidative stress level in the RGCs and by stimulation of the BDNF-TrkB pathway [19, 50]. In addition, VPA has been shown to exert antioxidant properties in the brain following ischemia/reperfusion injury [51] and in motor neurons following spinal cord injury [52]. Since VPA has been reported to increase activities of superoxide dismutase (SOD), catalase, and glutathione peroxidase in the retina following ischemia/reperfusion injury [53], it can be postulated that VPA acts on RGCs as an HDAC inhibitor resulting in increased expression of antioxidant enzymes such as SOD and catalase in glaucoma. Interestingly, clinical studies have reported that oral administration of VPA improves visual function in patients with retinitis pigmentosa, which is a group of hereditary eye disease that is characterized by selective degeneration of photoreceptors [54–56]. VPA is a drug that is already established for use in treatment of conditions other than retinal diseases, like epilepsy, with relatively minor side effects. Together with the data to indicate its therapeutic efficacy in glaucoma and retinitis pigmentosa, VPA is a suitable candidate for “drug repurposing,” which is an application of known drugs to new conditions. VPA is a promising therapeutic candidate for glaucoma and retinitis pigmentosa and further studies are required to assess its efficacy and safety for retinal diseases.

### 3.4. Spermidine

**Figure 1:**
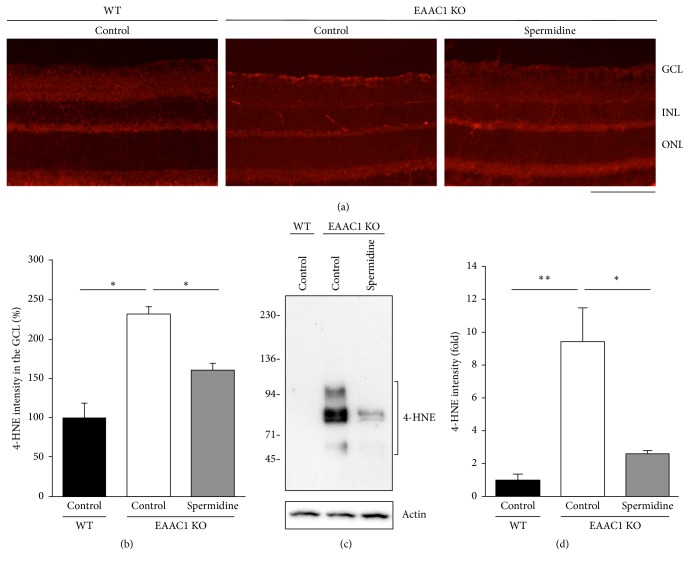
Spermidine reduces oxidative stress levels in the EAAC1 KO mouse retina. (a) Representative images of 4-HNE in the retina at 8 weeks old. Scale bar: 100 *μ*m. (b) Quantitative analyses of (a). Data are normalized to the 4-HNE intensity at the GCL in control WT mice (100%). *n* = 6 in each group. (c) Representative images of immunoblot analyses of 4-HNE in the retina at 8 weeks old. (d) Quantitative analyses of (c). Data are normalized to the 4-HNE intensity in control WT mice (1.0). *n* = 6 in each group. ^*∗∗*^*P* < 0.01; ^*∗*^*P* < 0.05. Reproduced from Noro et al. [20].

Spermidine is a naturally occurring polyamine that is essential for life. There is an association between decline of spermidine concentration and human aging, and exogenous application of spermidine extended the lifespan of yeast, flies, worms, and human immune cells by promoting autophagy, which leads to enhanced resistance to oxidative stress and decreased cell death [57]. Indeed, spermidine-treated yeast cells and mouse fibroblast cells are more resistant to damage induced by H_2_O_2_ treatment than nontreated cells, and feeding mice with spermidine increases the serum level of free thiol groups, indicating that spermidine reduces oxidative stress both in vitro and in vivo [57, 58]. We previously reported that spermidine prevents RGC death and visual impairment following ONI and in EAAC1 KO mice, by reducing oxidative stress levels in the retina (Figure 1) [20, 59]. Interestingly, spermidine inhibits activation of the ASK1-p38 pathway in RGCs and suppresses inducible nitric oxide synthase (iNOS) expression in microglia following ONI [59]. These findings indicate that oral intake of spermidine and its antioxidative effects are beneficial in treatment of glaucoma and traumatic optic neuropathy. Spermidine is a natural component of our diet and evidence shows that eating food that is rich in spermidine, such as soybeans and mushrooms, results in increased blood spermidine levels [60], suggesting that beneficial effects of spermidine can be easily attained by making a conscious choice of food.

### 3.5. Candesartan

**Figure 2:**
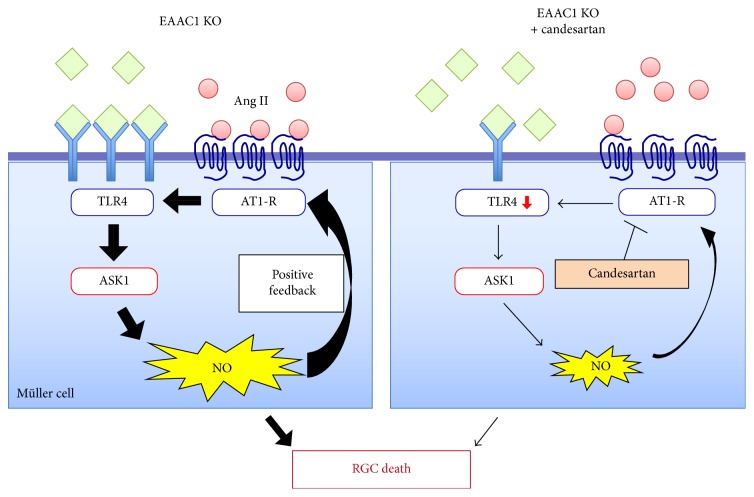
The proposed model of the effect of candesartan in EAAC1 KO mice. Increased oxidative stress in EAAC1 KO mice induces the upregulation of AT1-R and TLR4, resulting in increased NO expression via the ASK1 signalling pathway, which leads to RGC death. NO further stimulates AT1-R expression levels through a positive feedback loop. Candesartan blocks AT1-R and exerts neuroprotective effects by suppressing the upregulation of TLR4 and thus reducing ASK1-mediated NO production. This also results in inhibition of the positive feedback loop between NO and AT1-R. Reproduced from Semba et al., [17].

Candesartan is an angiotensin II receptor antagonist that is clinically used for treatment of hypertension. It modulates the renin-angiotensin system, which regulates the arterial blood pressure and thus plays a major role in the cardiovascular system. The renin-angiotensin system has been reported to be involved in oxidative stress-induced RGC death [61]. Indeed, we reported that suppression of the renin-angiotensin system by candesartan led to RGC protection and preservation of visual function in EAAC1 KO mice [17], suggesting that this drug may be a good drug repurposing candidate for glaucoma therapy. In the EAAC1 KO mouse retina, the expression of Toll-like receptor 4 (TLR4) is increased, which stimulates the ASK1 signalling pathway and upregulates iNOS expression in Müller glia leading to RGC death (Figure 2). Candesartan demonstrates neuroprotective effects by suppression of TLR4 upregulation in EAAC1 KO mice [17]. TLR4 polymorphisms are associated with NTG [62, 63]. Therefore, targeting TLR4 may be a promising strategy for treatment of glaucoma.

### 3.6. Nuclear Factor Erythroid 2-Related Factor 2 (Nrf2)

Nuclear factor erythroid 2-related factor 2 (Nrf2) is a transcription factor that is activated by oxidative stress and is a master regulator of various antioxidant pathways. Consequently, Nrf2 KO mice are susceptible to a wide range of toxicity and disease conditions associated with oxidative stress. Following ONI, RGC death is significantly increased in Nrf2 KO mice [64], and gene therapy with Nrf2 reduces RGC death [65]. In addition, agents such as *α*-lipoic acid and VPA exert neuroprotective effects by inhibition of ROS generation through activation of the Nrf2/HO-1 pathway [66, 67]. These findings suggest that activation of Nrf2 may be an effective therapeutic target for glaucoma.

## 4. Oxidative Stress and Optic Neuritis

In preclinical studies, experimental autoimmune encephalomyelitis (EAE), which is an animal model of MS, is often used to study optic neuritis. There are accumulating data that indicate oxidative stress plays a major role in the pathogenesis of MS and EAE [74]. Indeed, studies demonstrate that antioxidants are effective in suppressing inflammation in the optic nerve. For example, lipoic acid, a natural antioxidant, ameliorates inflammation and protects the optic nerve in EAE mice [75], and spermidine reduces oxidative stress in the optic nerve as well as in the RGCs in EAE mice leading to reduced optic nerve demyelination, RGC death, and visual impairment [70]. Another drug of interest is GGA, which reduces oxidative stress and is effective in protection of RGCs in glaucoma models as mentioned earlier. We previously reported that oral administration of GGA suppresses demyelination of the optic nerve, RGC death, and visual impairment in EAE mice [76], suggesting that GGA is a good therapeutic candidate for optic neuritis. Furthermore, gene therapy with antioxidant genes, namely,* SOD2* and* catalase*, was effective in reducing optic nerve demyelination, axonal loss, and RGC loss in EAE mice [77, 78]. These findings suggest that oxidative stress is associated with the pathogenesis of optic neuritis and is an effective target for its treatment.

## 5. Inhibition of Oxidative Stress for Treatment of Optic Neuritis

The summary of this section is shown in Table 1.

### 5.1. ASK1 and Optic Neuritis

In addition to immune cells such as T cells and dendritic cells, glial cells play important roles in demyelinating neuroinflammation [79, 80]. Indeed, the ASK1-p38 pathway in astrocytes and microglia plays essential roles in release of key cytokines including monocyte chemoattractant protein-1 (MCP-1), macrophage inflammatory protein-1 alpha (MIP-1*α*), regulated on activation, normal T cell expressed and secreted (RANTES), and TNF-*α* during neuroinflammation [37]. In EAE ASK1 KO mice, reduction of such proinflammatory cytokines as well as decrease in upregulation of iNOS leads to suppression of neuroinflammation and demyelination of the optic nerve, suggesting oxidative stress plays a part in degeneration of the optic nerve in this model [37]. Since VPA ameliorates inflammation of the spinal cord in EAE mice by suppressing the activation of T cells [81], we applied VPA to EAE ASK1 KO mice and found that VPA and ASK1 inhibition have synergistic therapeutic effects during EAE [69]. In addition, EAE induces reduction in visual function, which can be assessed by electroretinogram, but ASK1 deficiency ameliorates this visual impairment [37], suggesting that inhibition of ASK1 is effective both histologically and functionally. We have previously demonstrated that oral administration of an ASK1 inhibitor, MSC2032964A, is effective in suppressing neuroinflammation and demyelination in EAE mice [37]. These results suggest that inhibition of ASK1 is a promising strategy for treatment of optic neuritis. In fact, suppression of oxidative stress with inhibition of the ASK1 activity holds therapeutic potential for various neurodegenerative diseases such as MS and glaucoma [68].

### 5.2. Brimonidine

Brimonidine is an *α*_2_-adrenergic receptor agonist that is clinically used to lower IOP in glaucoma patients. Recent studies indicated that the therapeutic effect of brimonidine does not solely depend on reducing IOP. For example, brimonidine increases cultured RGC survival from oxidative stress damage [71], it increases glial expression of neurotrophic factors that are important for RGC survival and decreases phosphorylation of the GluN2B subunit in the retina, thereby reducing activation of the NMDA receptors leading to reduced RGC death [18]. These data suggest that pharmacological actions of brimonidine may include suppression of oxidative stress directly and indirectly. In EAE mice, daily treatment with brimonidine eyedrops led to reduction in RGC death and visual function, suggesting that brimonidine is an effective agent for preventing RGC loss and visual impairment in optic neuritis [73]. In this study, brimonidine eyedrops did not have any effect on demyelination of the optic nerve, but this may not be surprising as the route of administration was topical, directly to the eye. Since brimonidine exerts neuroprotective effects against glutamate excitotoxicity-induced oxidative stress [72] and oligodendrocytes in the optic nerve are vulnerable to glutamate neurotoxicity [82], it would be interesting to investigate if brimonidine can indeed prevent demyelination if administered* via* a different route, such as systemically or directly into the optic canal.

## 6. Conclusions

Oxidative stress plays an important part in the pathogenesis of neurodegenerative disease and neuroinflammation. Furthermore, increased oxidative stress is associated with aging [83] and with drastic increase in life expectancy worldwide [84], there is an urgent need to cure or manage age-related chronic neurodegenerative conditions such as glaucoma, Alzheimer's disease, and Parkinson's disease. Currently, established treatment for glaucoma and optic neuritis does not involve targeting oxidative stress. However, preclinical data indicate that suppression of oxidative stress is a promising strategy for many eye diseases including glaucoma and optic neuritis.

## Figures and Tables

**Table 1:** Possible genes and drugs targeting oxidative stress for the treatment of glaucoma and optic neuritis.

Therapeutic target	Glaucoma	Optic neuritis
ASK1	References [15, 35, 36, 68]	References [37, 68, 69]
Dock3	References [16, 42, 43, 47]	
VPA	References [19, 50, 53]	Reference [69]
Spermidine	References [20, 59]	Reference [70]
Candesartan	References [17, 61]	
Nrf2	References [64–66]	
Brimonidine	References [18, 71, 72]	Reference [73]
